# A Long Life Moisture‐Enabled Electric Generator Based on Ionic Diode Rectification and Electrode Chemistry Regulation

**DOI:** 10.1002/advs.202305530

**Published:** 2024-02-14

**Authors:** Chunqiao Fu, Jian Zhou, Xulei Lu, Haochen Feng, Yong Zhang, Kedong Shang, Zhongbao Jiang, Yuming Yao, Qi‐Chang He, Tingting Yang

**Affiliations:** ^1^ Tribology Research Institute School of Mechanical Engineering Southwest Jiaotong University Chengdu 610031 P. R. China; ^2^ Univ Gustave Eiffel MSME CNRS UMR 8208 Marne‐la‐Vallée F‐77454 France

**Keywords:** electrode chemistry regulation, hydrovoltaic, ionic diode, long lifetime, moisture

## Abstract

Considerable efforts have recently been made to augment the power density of moisture‐enabled electric generators. However, due to the unsustainable ion/water molecule concentration gradients, the ion‐directed transport gradually diminishes, which largely affects the operating lifetime and energy efficiency of generators. This work introduces an electrode chemistry regulation strategy into the ionic diode‐type energy conversion structure, which demonstrates 1240 h power generation in ambient humidity. The electrode chemical regulation can be achieved by adding Cl^−^. The purpose is to destroy the passivation film on the electrode interface and provide a continuous path for ion‐electron coupling conduction. Moreover, this device simultaneously satisfies the requirements of fast trapping of moisture molecules, high rectification ratio transport of ions, and sustained ion‐to‐electron current conversion. A single device can deliver an open‐circuit voltage of about 1 V and a peak short‐circuit current density of 350 µA cm^−2^. Finally, the first‐principle calculations are carried out to reveal the mechanism by which the electrode surface chemistry affects the power generation performance.

## Graphical abstract

A moisture‐enabled electric generator for power generation at high humidity for 1240 h is presented. The device simultaneously meets the requirements of fast capture of moisture molecules, high rectification ratio transport of ions, and sustained ion‐electron current conversion, and also provides a reliable solution for ultra‐long‐time humidity power generation.

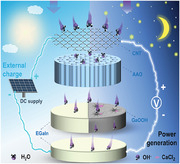

## 1 Introduction

Traditional renewable energy sources, such as solar, wind, geothermal, and biomass,^[^ ^1^ ^]^ have strict requirements for production sites and climatic conditions.^[^ ^2^ ^]^ Hydrovoltaic power generation, which uses water interacting with functional materials to generate electricity directly,^[^ ^3^ ^]^ is more flexible and can complement the advantages of traditional renewable energy sources.^[^ ^4^ ^]^ Early hydrovoltaic power generation, mostly around droplets,^[^ ^5^ ^]^ waves,^[^ ^6^ ^]^ and water evaporation,^[^ ^7^ ^]^ has achieved rapid development. Moisture, as an important part of the natural water cycle process, can be a carrier of thermal, mechanical, and chemical energy. Using low‐dimensional nanostructures to capture energy in moisture, the realization of large‐scale ionization, and rapid transport of ions to generate electricity are expected to contribute to solving the growing energy demand and energy crisis in the future.^[^ ^8^ ^]^ In recent years, material systems for hygroelectric generation have evolved from amorphous carbon black,^[^ ^9^ ^]^ graphene oxide (GO),^[^ ^10^ ^]^ carbon nanotubes (CNT),^[^ ^11^ ^]^ and other carbon‐based materials to polyelectrolytes,^[^ ^12^ ^]^ biomaterials,^[^ ^13^ ^]^ inorganic oxides,^[^ ^1a^ ^]^ and so on.

Numerous works dedicated to generating moisture‐based power can be roughly classified into three categories according to the three models described now. The first one, underlying the majority of moisture‐based power generation devices, relies on ion concentration gradients. Three common gradients, that is, the initial functional group distribution gradient of nanostructured materials,^[^ ^14^ ^]^ the moisture adsorption gradient,^[^ ^15^ ^]^ and the hygroscopic ion distribution gradient,^[^ ^16^ ^]^ lead to ion diffusion processes upon the action of water molecules.^[^ ^17^ ^]^ This type of power generation has received large attention and the produced transient power density can be very high (even up to 103.1 MW cm^−2^).^[^ ^18^ ^]^ However, the migration of ions may slow down quickly and reach an equilibrium state where power generation ceases. The reported power generation duration of such devices varies typically from seconds to days.^[^ ^7^, ^14^, ^19^ ^]^ In search of a more generalized approach to generator design, researchers have begun to integrate and explore a variety of models, such as Air gen, which was designed through the combination of hygroscopic gradient and capacitance principles and can be applied to moisture‐enabled power generation of a wide range of nanomaterials.^[^ ^20^ ^]^

The second model of moisture‐based power generation exploits the phenomenon of moisture fluctuations to continuously supply energy. Typically, ionic droplets are placed on the surface of charged nanostructured materials.^[^ ^21^ ^]^ When moisture increases, the ionic droplets tend to absorb water and expand continuously, while the nanostructure pegging prevents the advance of the contact line. This leads to the flow of the ionic liquid from the edge to the center so as to remove the surrounding excess fluid, producing a relatively stable potential difference at the solid/liquid interface. In contrast to the bottom‐inward flow caused by moisture absorption, the dehumidification process reverses the flow direction of droplets for a similar reason. Thus, both moisture absorption and dehumidification can generate electricity, and their output voltages are in the opposite direction. Moreover, theoretically speaking, as long as the moisture absorption and dehumidification process exists, ions can continue to flow and generate electricity. However, the power output of this type of device depends largely on the speed of moisture absorption and desorption. The air humidity changes slowly in most scenes on the earth, which limits output power.

The third model of moisture‐based power generation, typically underlying the nanofluidic diode device, is inspired by the photovoltaic effect.^[^ ^22^ ^]^ A PN junction is formed by contacting a negatively charged nanofluidic channel with a positively charged nanopore. When moisture is adsorbed on the upper surface of the nanochannel, liquid water is formed due to the capillary coalescence effect, and heat energy is released. The water molecule ionizes protons and anions under the action of thermal energy and the built‐in electric field of the PN junction. The anions and cations then achieve reverse directional migration under the action of the built‐in electric field and gather at both ends of the electrodes to generate a stable potential difference. As more and more ions migrate, the ions gathered near the electrodes inevitably hinder the continued migration of subsequent ions. To address this problem, active metal electrodes are introduced to continuously consume the ions accumulated at both ends through redox reactions, making the life of the device break through to at least 1 month. However, the metal electrode of the device rapidly forms a dense passivation film during the oxidation process, blocking the ion transport path. The current density decays gradually from 1.5  to 0.1 µA cm^−2^ within 1 month of continuous power generation.

**Figure 1:**
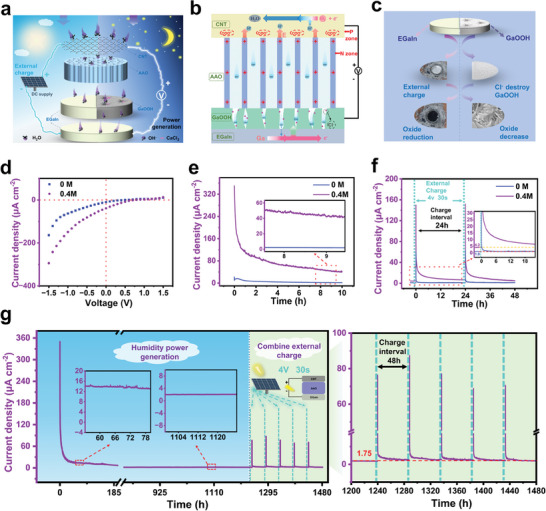
a) Schematic diagram of MEG structure. b) Schematic diagram of MEG working principle. c) Two strategies of electrode passivation layer suppression. d–f) MEG with and without calcium chloride: respective *I*–*V* curve comparison, current density comparison, and charge/discharge output current density curve comparison. g) Ultra‐long operation curve of a single device under coupling mode of hygroelectricity and photovoltaic charging.

In this work, we introduce a small amount of calcium chloride into a nanofluidic diode device, which greatly improves the output power and lifetime. According to experiments and first‐principles calculations, calcium chloride gives rise to four advantageous features. First, due to the adsorption of chloride ions on oxygen atoms, the generated passivation layer is destroyed. Thus, OH^−^ is smoothly moved to the vicinity of the lower electrode, completing charge conversion to supply continuous power output, improving the working life of the device. Second, an appropriate amount of ion addition increases the rectification ratio of the diode device and improves the separation efficiency of anions and cations. Therefore, the internal energy loss of the device is reduced so as to optimize the device output power. Third, calcium chloride has water absorption properties and can effectively capture moisture. Moreover, an increase in the number of water molecules and ions in the nanopores reduces the internal resistance of the device, improving energy conversion efficiency and also augmenting the device output power. Fourth, the addition of Cl^−^ also promotes the reduction of the electrode passivation layer and enables the passivation layer to be self‐eliminated during external charging. Therefore, our fabricated device shows 1240 h of power generation operation under a 93% RH humid environment. This novel way of combining hygroelectric generation and charging also provides a path to the design of a full‐time power generation system based on the humidity‐optical coupling principle. During the day, photovoltaic power generation is dominant and a small amount of electricity is used to activate the hygroelectric module; during the night the moisture‐enabled electric generator (MEG) is favored, as shown in **Figure** **1**a.

## 2 Results and Discussion

### 2.1 Device Structure Design and Fabrication

Here, we used an ionic diode hybrid membrane to fabricate a long‐time MEG. The device structure consists of a CNT membrane, an anodized aluminum oxide (AAO) membrane, and a metal lower electrode. Among them, the nanoporous CNT membrane was prepared by the chemical vapor deposition (CVD) method, and the diameter of the tube bundle is mostly distributed between 10 and 30 nm, and the thickness is 120–140 nm. The CNT membrane has a large number of micro‐nano pores to facilitate the transport of water molecules and ions. After treatment with oxygen plasma, its surface is rich in oxygen‐containing functional groups (Figure S1, Supporting Information). The functional groups on CNT ionize a large number of protons in the presence of water molecules, leaving the negative surface charge that serves as the P‐region of the nanofluidic diode. The negative potential at the surface of the CNT film was also illustrated using the KPFM test, and when the CNT was loaded on the device, the surface potential was positive, which also corresponded to the positive pole of the device (Figure S2, Supporting Information). The AAO is endowed with uniform nanochannels with a pore size of 90 nm and a large positive charge on its surface (Figure S3, Supporting Information), which acts as the N‐region of the nanofluidic diode. The lower electrode is made of eutectic gallium‐indium (EGaIn), which exhibits excellent conductivity, hydrophilicity, and activity, while its liquid property allows it to combine well with AAO. The main difference from previous work is that an appropriate amount of calcium chloride solution^[^ ^22c^ ^]^ was deposited by spin coating on the lower surface of AAO far away from the PN junction region (Figure S4, Supporting Information). The distribution of the corresponding ions in the channel is shown in Figure S5, Supporting Information. There is no difference in the distribution of calcium ions and chloride ions in the AAO channel, indicating that the device shows no selectivity for chloride ions and calcium ions; thus, the device will not generate electricity due to the salinity difference between the upper and lower electrodes. One of the benefits brought by the addition of calcium chloride is that it can effectively capture water molecules in the environment (Figure S6, Supporting Information). The increase in the number of water molecules and ions in the nanopores reduces the internal resistance of the device and improves its conductivity (Figure S7, Supporting Information).

To better illustrate the innovative idea of this paper, the power generation principle of the previous work is first introduced in detail, as shown in Figure 1b. There is a built‐in electric field pointing from the N region to the P region in the CNT/AAO interface region. Due to the interaction of nanopores and water molecules, hydrogen ions and hydroxide ions are ionized. Under the action of the built‐in electric field, OH^−^ moves to the lower electrode in the direction opposite to the electric field, while H^+^ moves to the upper electrode in the direction of the electric field. With the continuous action of moisture, the H^+^ and OH^−^ accumulated near the electrode are eventually consumed by the redox reaction, which converts the ionic current into electronic current and thus achieves an external power supply.

We believe that spatial separation of ions is the basis for power generation. In order to verify the above viewpoint, we did AAO replacement experiments (Figure S8, Supporting Information). The negatively charged silica nanopore film and the uncharged titanium dioxide nanopore film were used to replace the AAO (positively charged), and the as‐fabricated devices were tested. The experiments have demonstrated that the antisymmetric surface polarity of nanopores is a prerequisite for high‐performance power generation. Even if the electrode is made of active metal, if the ion diode structure is not met, the device will still have no power output. We also did humidity gradient verification experiments, with specific reference to Figure S9, Supporting Information. By sealing or not sealing the lower electrode, we proved that the humidity gradient between the upper and lower electrodes is also an important influence on the power generation performance. Since the spatial separation of ions needs energy, this part of the energy we think comes from the hydrovoltaic effect, which specifically should include the humidity gradient energy and the thermal energy released by the gas‐liquid phase transition. At the same time, the introduction of the redox reaction is essential for power generation. On the one hand, it continuously consumes the ions enriched near the electrode, clearing the obstacles for the continuous migration of subsequent ions, and on the other hand, it converts the ionic charge into an electronic charge to realize the external power supply.

Since gallium is more active than indium, the redox reaction at the electrode can be expressed as follows

At the negative (anode) electrode

(1) $$\mathrm{Ga}+3OH^{-}⇄\mathrm{GaOOH}+H_{2}O\;+\;3e^{-}$$

At the positive (cathode) electrode:

(2) $$O_{2}+4H^{+}+4e^{-}\to 2H_{2}O$$

As shown in Equation (1), when the reaction continues, a dense oxide passivation layer GaOOH is formed on the lower electrode surface, which hinders the subsequent OH^−^ ion migration and weakens the redox reaction, and the power generation performance of the device gradually decays. At high humidity of 93% RH, the short‐circuit current density of the device gradually decreases from 1.5 µA cm^−2^ to about 0.1 µA cm^−2^, although the device can continue to generate electricity for at least 1 month.^[^ ^22c^ ^]^ To demonstrate that O_2_ in the reaction Equation (2) is actually involved in the entire reaction, we used alternating streams of O_2_ and the inert gas N_2_. The decay of the current in the N_2_ environment contrasts markedly with the rapid rise evident in the O_2_ environment, illustrating the irreplaceable role of O_2_ in the energy generation process (Figure S10, Supporting Information).

In the present work, we find that the calcium chloride additive plays a key role in the substantial improvement of the device power generation and operating lifetime, mainly stemming from its destruction of the passivation layer (Figure 1c) and optimization of the ionic rectification effect (Figure 1d). The specific mechanism is described and analyzed in detail in the later section. In addition to the calcium chloride strategy, the application of a voltage to charge the device after a period of operation also facilitates the rapid reduction of the oxide passivation layer on the electrode surface, as shown in Figure 1c. Figure 1e shows that the current density of the calcium chloride‐doped device is about 25 times higher than that of the untreated sample and the output voltage is increased by 10% after 10 h of continuous discharge at 93% RH (Figure S11, Supporting Information).

Moreover, the addition of calcium chloride also improves the rechargeable performance of the device, as displayed in Figure 1f. Under a short time (30 s) 4 V voltage loading, the current density of the device without additive drops to the pre‐charge level after about 4 h, and the current density of the device with an additive is 6.35 µA cm^−2^ even after 24 h, which is still higher than the 4.2 µA cm^−2^ before charging. The design also provides a possible future mode of photovoltaic humidity‐coupled power generation, where a small amount of energy from the midday photovoltaic power generation is used to activate the passivation layer, resulting in significantly improved moisture‐based power generation performance (Figure 1g).

### 2.2 Improvement of Rectification Ratio and Destruction of Passivation Layer by Calcium Chloride Additive

**Figure 2:**
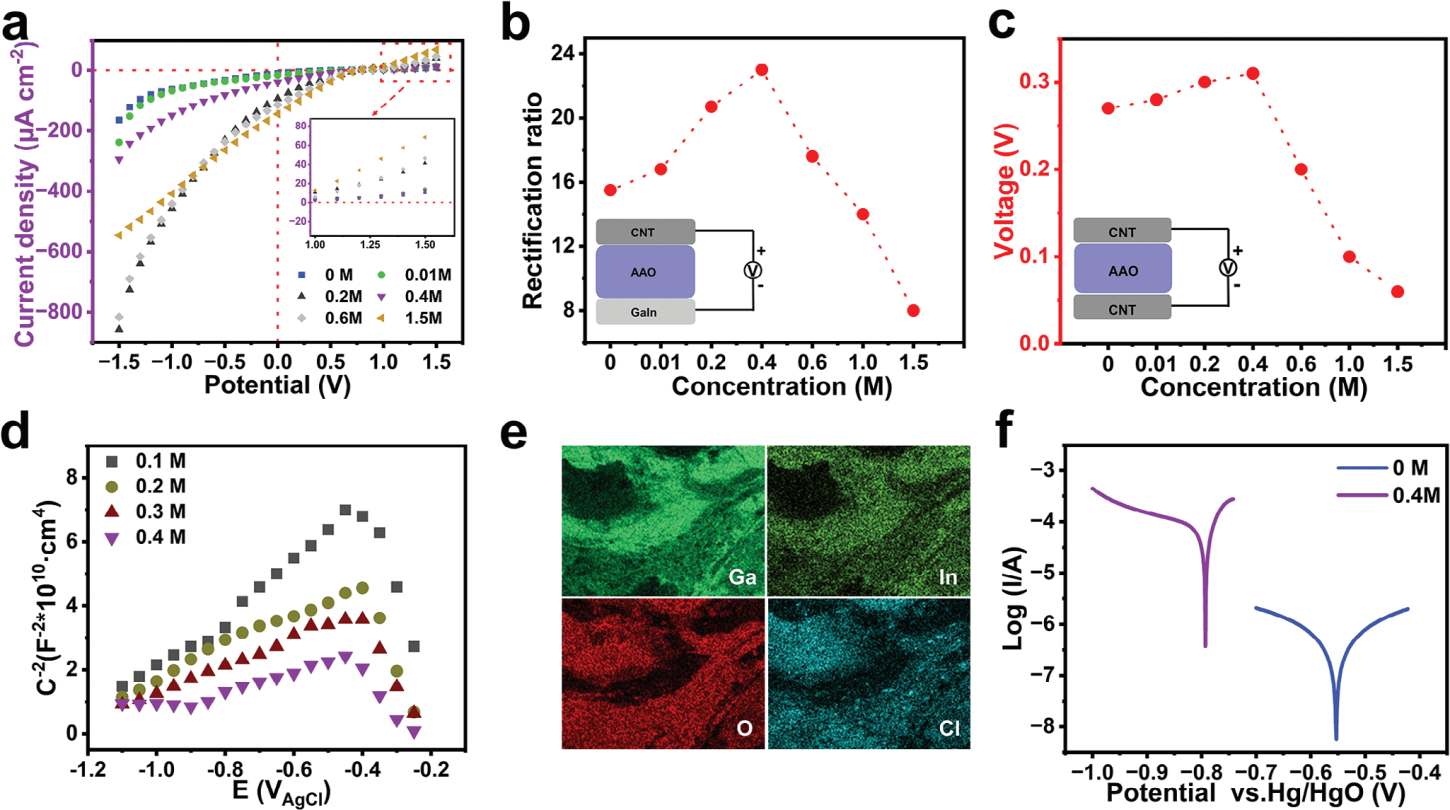
a) Ionic *I*–*V* curve of MEGs with nanofluidic diode effect under different concentrations of additives. b) MEG rectification ratio values under different concentrations of additives. c) Open circuit voltage of devices with double carbon material electrodes at different concentrations of additives. d) Mott–Schottky plots of the passive films formed on the EGaIn electrode with different concentrations of additives. e) EDS mapping of EGaIn electrode oxide. f) Tafel plots of EGaIn electrode in different concentrations of additives.

First, the ion‐directed migration behavior of the ion diode is studied by current–voltage (*I*–*V*) measurements. As shown in **Figure** **2**a, the *I*–*V* curve is used to scan from negative voltage to positive voltage direction. The *I*–*V* curve shows the non‐ohmic characteristics of the device, indicating that the device has ion rectification features. At the positive bias, the applied bias voltage is opposite to the built‐in electric field formed at the CNT‐AAO interface. This weakens the built‐in electric field and reduces the enrichment of ions at the positive and negative electrodes, leading to a lower redox reaction rate and corresponding to a lower current density. Under the reverse voltage, the applied bias voltage is in the same direction as the built‐in electric field formed at the CNT‐AAO interface, which enhances the built‐in electric field. As a result, the generation rate of hydrated charged ions increases, ions are enriched near the electrode, the redox reaction rate augments, and the corresponding current density is intensified.

In order to quantify the effect of the additive concentration on the rectification ratio of the diode, the device currents at −1.5 and +1.5 V are calculated to obtain the rectification ratio, which is used to evaluate the ability of the diode to regulate the directional migration of ions. As shown in Figure 2b, the rectification ratio of the device without additive is 15.5, indicating that the device already has a rectifying effect without additive, due to the built‐in electric field formed between CNT and AAO. At low concentrations, the rectification ratio is low due to the concentration polarization occurring at the entrances of the nanopores. As the concentration increases, the concentration polarization weakens and the rectification ratio keeps increasing. The rectification ratio reaches a maximum value of 23 when the concentration is equal to 0.4 m (2 uL). As the concentration continues to enlarge, the EDL thickness decreases and the rectification ratio gradually diminishes. This trend is consistent with previously reported diodes.^[^ ^23^ ^]^

The spatial separation of anions and cations constitutes the basis for device power generation so the rectification effect should be closely related to the power generation performance. To check if this is true, the open circuit voltage performance of MEG devices under different concentrations of calcium chloride additives (corresponding to different rectification ratios) has been investigated. To avoid the interference of the redox reaction of metal electrodes, the lower electrode of EGaIn is replaced with a chemically inert CNT electrode to test the device's open‐circuit voltage (Figure 2c). In the 93% RH environment, there is an obvious voltage signal in the device, indicating that the built‐in electric field of the diode successfully drives the reverse directional transport of anions and cations. The open‐circuit voltage is 0.27 V in the absence of additive and gradually augments with increasing additive concentration, reaching a maximum value of 0.31 V at an ion concentration of 0.4 m. As the concentration continues to increase, the open‐circuit voltage starts to decrease. The dependence trend of the open‐circuit voltage on the additive concentration tends to coincide exactly with the trend of the rectification effect, which further proves that the rectification effect is the basis of device power generation. In order to optimize ion selectivity and ion transmission capabilities, follow‐up studies uniformly select 0.4 m calcium chloride additive (2 uL).

According to the principle of metal passivation, anions exert a stronger destructive effect on the passivation film.^[^ ^24^ ^]^ To demonstrate the effectiveness of chloride ions, we used different chloride and calcium ion compounds for output performance tests (Figure S12, Supporting Information). The experimental results show that the device performance is dominated by chloride ions. The metal passivation film has semiconductor properties and its formation and rupture depend on the transport process of electrons and ions. Therefore, the active state of the passivation film is closely related to its semiconducting electronic properties. The semiconducting parameters of the passivation film were tested at different concentrations of chloride ions. Figure 2d provides the capacitance measurements of the passivated films for four sets of additive concentrations ranging from 0.1 to 0.4 m. The slope of the linear segment is positive, implying that the passivation film exhibits n‐type semiconductor properties.

According to the equation derived from the Mott–Schottky model, the capacitance C^−2^ varies linearly with the applied electrode potential E for n‐type semiconductors.^[^ ^25^ ^]^ The passivation film is composed of GaOOH and *N*_D_ corresponds to the defects in the passivation film; the donor density *N*_D_ of the passivation film can be estimated from the slope of the linearly fitted Mott–Schottky plot according to the following equation

(3) $$C^{-2}=\frac{2}{\varepsilon \varepsilon_{0}eN_{D}}\;\left(E-E_{\mathrm{FB}}-\frac{KT}{e}\right)$$

where *C* is the space charge layer capacitance, *e* stands for the electron charge (1.6 × 10^−19^C), *ɛ* corresponds to the dielectric constant of the passivation film, *ɛ*_0_ symbolizes the vacuum dielectric constant (8.854 × 10^−14^ F cm^−1^), *k* represents the Boltzmann constant (1.38 × 10^−23^ J K^−1^), and *T* means the absolute temperature (K). Table S1, Supporting Information, shows the defect density *N*_D_ in the passivation film, which is calculated from the slope of the curve (Figure 2d) and Equation (3). As the chloride ion concentration increases, the donor density *N*_D_ augments, with the highest *N*_D_ equal to 0.496 for the passivation film with a concentration of 0.4 m. This indicates that chloride ions can effectively destroy the oxide film of EGaIn, bringing about more film defects to provide paths for ion transport.

According to the point defect model (PDM) theory, the possible defects of n‐type semiconductor passivation film are oxygen vacancies.^[^ ^26^ ^]^ The destructive effect of chloride ions is mainly due to the adsorption and substitution of chloride ions on the oxygen atom groups inside the oxide. To check this conjecture, the oxides were first characterized by XPS (Figure S13, Supporting Information),^[^ ^27^ ^]^ and subsequently, energy dispersive X‐ray spectroscopy (EDS) component analysis of the oxide was performed. It can be seen from Figure 2e that the positions of oxygen and chlorine on the oxide are highly overlapped, indicating that chlorine ions have an adsorption effect on oxygen atoms. Moreover, the adsorption and replacement of oxygen by chloride ions are also illustrated by comparing the changes in oxygen content with and without calcium chloride additive (Figure S14, Supporting Information). Subsequent theoretical calculations also simulate the damage behavior of chloride ions on the passivation film; refer to Section 2.4.

The addition of chloride ions destroys the passivation layer and theoretically favors the improvement of the electrode activity. To verify the above conjecture, Tafel measurements (Figure 2f) were used to study the corrosion potential of the electrode at different concentrations of additives. Higher potential means less self‐discharge and lower activity.^[^ ^28^ ^]^ The results show that the potential at 0.4 m is −0.793 V, which is lower than −0.553 V without additives, and the potential decreases with increasing additive content, which strongly suggests that the presence of chloride ions improves the activity of the electrode.

### 2.3 Influencing Factors of Device Power Generation Performance

**Figure 3:**
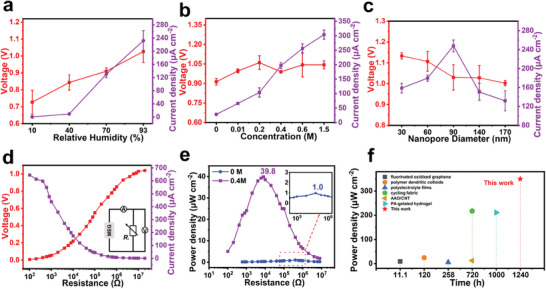
a) Effect of relative humidity on device performance. b) Device performance corresponding to different additive concentrations under moisture conditions. c) Effect of nanopore size on device performance. d) *V*_OC_ and *J*_SC_ changes with different external load resistances for the device with a working area of 0.28 cm^2^, the inset being the actual test circuit diagram. e) The corresponding output power density varies with the load resistance. When the external resistance is 10 kΩ, the device has the best output performance. f) Performance comparison between the MEG device and some recently reported moisture‐enabled electric generators.

The output performance of the device under different relative humidity degrees was investigated and shown in **Figure** **3**a. As the relative humidity rises from 10% to 93%, the *V*_OC_ increases monotonically from 0.72 to 1.02 V and does the *I*_SC_ monotonically from 0.42 to 233.32 µA cm^−2^. The increases in voltage and current density are mainly attributed to the increase in the number of free‐moving ions under high humidity conditions, leading to an augmentation of the number of ions involved in the rectification effect and redox reaction. The output performance of the device under different Calcium chloride concentrations was then investigated. As shown in Figure 3b, the increase in Calcium chloride concentration enhances the reactant activity at the electrode‐electrolyte interface, increasing the current density output and redox potential (*V*_REDOX_) at the electrode–electrolyte interface. The built‐in potential (*V*_B_) is mainly generated by the rectification effect of the ion diode. When the ion concentration is too large, the rectification effect becomes weak and *V*_B_ shows a downward trend, but the total output voltage (*V*_REDOX_+*V*_B_) remains basically stable. The *V*_OC_ fluctuates normally at ≈1 V. When the concentration exceeds 0.4 m, the weakening of the ion selectivity of the diode diminishes the contribution of the rectification effect to the power generation. This point will not be discussed in depth here.

The effect of the pore size of the AAO nanochannel on the device performance is shown in Figure 3c. The Debye length is related to the ion concentration in the solution and is usually equal to 1–100 nm. The repulsive enrichment effect of ions requires the minimum cross‐sectional size in the nanopore channel to be equal to or smaller than the Debye length,^[^ ^29^ ^]^ so that an excessively large pore size is not conducive to ion rectification. Moreover, due to the increase in pore size, the increase of adsorbable water molecules leads to a decrease in the effective concentration of the additive and thus a decrease in the electrode activity. Under the combined effect of the above factors, the power generation performance of devices with too large pore size is poor. On the other hand, too small pore size relatively limits water molecules captured by nanopores that can be dissociated, which reduces the number of ions involved in rectification and redox reactions.

The experimental results show that it is more appropriate to control the pore size around 90 nm. Figure 3d,e shows the power output performance of the device with a working area of 0.28 cm^2^ under applied load conditions. The optimal resistive load is 10 KΩ, which results in an output power density of 39.8 µW cm^−2^. This is a nearly 40‐fold increase compared to the loading power without the calcium chloride additive (1 µW cm^−2^), further highlighting the effectiveness of the calcium chloride additive strategy. In order to understand the level of our device performance, the maximum power (*P*_max_ = *V*_max_ × *J*_max_) calculation method compares the duration and performance of similar devices reported in the literature, as shown in Figure 3f and Table S2, Supporting Information. It follows from the comparison that most of the existing devices are unable to balance power generation time and output performance, while our MEG device has both excellent output power and long power generation time.

### 2.4 First‐Principle Simulation of Passivation Film Destruction Process

The first‐principle calculations were performed using the VASP software. A (3 × 3) GaOOH (001) supercell was used for computing the structural relaxation and electronic properties. The specific setting parameters are specified in the supplementary materials.

**Figure 4:**
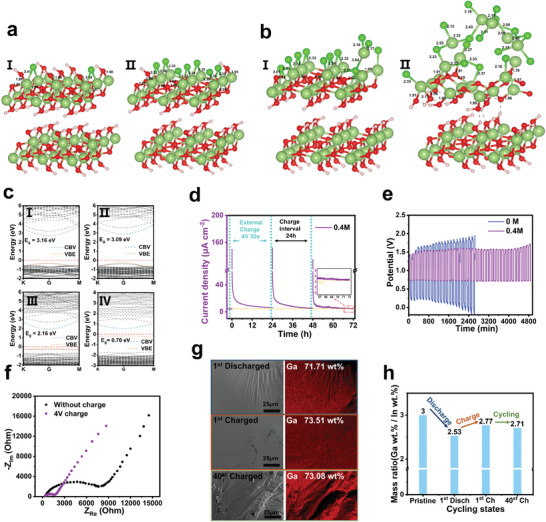
a) The adsorbed structures of Cl^−^ on the surface of GaOOH(001) at the coverage of 22.2% (aI), 77.8% (aII). Greenish atoms, hydrogen; Dark green, gallium; Pink, hydroxyl; Dark red, oxygen. b) The inserted structures of Cl^−^ into GaOOH(001) surface at the coverage of 22.2% (bI), 77.8% (bII). c) Electronic band structure plots of GaOOH(001) surface at the Cl^−^ adsorption coverage of 22.2% (cI) and 77.8% (cII), and at the Cl^−^ insertion coverage of 22.2% (cIII) and 77.8% (cIV). d) Short‐circuit current density of the device during short‐time charging and follow‐up discharging, the illustration on the right is the enlarged current density diagram. e) Long‐term cycling performance of devices with different additive concentrations at 1 µA cm^−2^. f) Comparison of device Nyquist curves before and after charging. g) EDS mapping image of EGaIn under different cycle conditions. h) Mass ratio of EGaIn in different cyclic states.

The initial step in the interaction of chloride with hydroxylated oxide surfaces is thought to involve competitive adsorption of chloride and OH^−^ at the oxide solution interface.^[^ ^30^ ^]^ We examined the adsorption behavior of Cl^−^ by substitution. The adsorption of Cl^−^ on the GaOOH (001) surface is achieved by replacing the OH^−^ on the surface with Cl^−^. There are nine exposed OH^−^ in the outermost layer that can be substituted, and these OH^−^ are gradually replaced by Cl^−^ as the Cl^−^ coverage increases. Here, we chose to reflect the change in GaOOH with increasing Cl^−^ coverage when two and seven OH^−^ are replaced, and the adsorption results are shown in **Figure** **4**a. When two OH^−^ are replaced (Figure 4aI), the Cl^−^ coverage is low (22.2%) and adsorption just starts. The bond length between Cl^−^ and Ga is 2.38 Å, which is higher than the experimentally observed GaCl_3_ bond length of 2.001 Å,^[^ ^31^ ^]^ indicating that the surface coordination bonds are weaker than the intramolecular chemical bonds. The reduced number of Ga─O bonds compared to the pristine GaOOH surface weakens the bonding between the first layer of Ga ions and O ions. When seven OH^−^ are substituted (Figure 4aII), the coverage of Cl^−^ is high (77.8%). The distance between the top Ga ion and the inner O ion increases slightly from 2.07 to 2.08 Å, indicating that the binding between the top and inner layers is reduced by the adsorption of Cl^−^, but this effect is not significant. Therefore, there is no obvious ion layer splitting in GaOOH during Cl^−^ adsorption, implying that the changes brought by increasing Cl^−^ adsorption concentration to GaOOH are limited.

We studied the ion exchange behavior by a Cl^−^ insertion model and simulated Cl^−^ insertion by exchanging the positions between Cl^−^ and the closest O ion in the first inner layer of the GaOOH membrane, based on the fact that all OH^−^ in the outermost layer of the surface is replaced by Cl^−^ (whose coverage is 100%). The oxygen ions have 16 different positions in the first inner layer, and placing Cl^−^ inside increases the concentration of insertion. We chose the substitution of two and seven oxygen ions in the inner layer to show the variation of GaOOH with concentration caused by Cl^−^ insertion, and the relaxation results are shown in Figure 4b. As Cl^−^ insertion proceeds, relaxation occurs not only in the inner layer but also in the outermost chloride layer. When the two oxygen ions of the inner layer were replaced (Figure 4bI), the Cl^−^ insertion had just started and the interlayer distance between the inserted layer and the lower layer was 1.617 Å. Notably, a significant relaxation occurred in one of the outermost Ga ions, indicating that the Cl^−^ insertion might cause damage to the surface layer of GaOOH.

When the seven oxygen ions in the inner layer were replaced (Figure 4bII), the number of Ga─O bonds in the inner layer was greatly reduced, the outermost Ga ion underwent collective relaxation and deviated from its original position, and the distance between the inserted and lower layers became 1.106 Å. At this time, the Cl^−^ inserted into the inner layer also escaped to the outermost layer, and due to the loss of Ga─O bonds at high concentrations, the original stable structure of the Ga ion layer became difficult to maintain. Therefore, with the increase of Cl^−^ insertion concentration, the outermost surface becomes more unstable, and the top layer which loses the Ga─O bond may transfer the formed chloride into the solution at high concentration, further exposing the new surface and leading to the rupture and thinning of the passivation film.

We performed calculations on the band structure of the four cases mentioned above and presented them in Figure 4c. The initial value of the band gap is 3.02 eV (Figure S15, Supporting Information). With the increasing coverage of Cl^−^ adsorption (Figure 4cI,II), the bandgap initially augments and then decreases, while the material itself undergoes no significant change. This can be attributed to the competition between Cl^−^ and OH^−^ for adsorption sites, which leads to a redistribution of surface charges. As Cl^−^ ions are inserted into the film (Figure 4cIII,IV), the bandgap starts to decrease and continues to do so with increasing insertion concentration. The significant decrease in bandgap (≈0.7 eV) is mainly caused by the increased concentration of defects resulting from the structural relaxation induced by Cl^−^ insertion. This greatly enhances the electrical conductivity of GaOOH films and reduces their structural stability.

### 2.5 Reduction of the Passivation Layer by Charging

Gallium‐indium alloy, as a liquid metal, has a certain mobility and the surface oxide film can be reduced under the applied voltage (Video S1, Supporting Information);^[^ ^32^ ^]^ based on this special reduction property, the repeated charging and discharging of the device was explored. The gallium‐indium alloy electrode was used as the negative electrode and the CNT as the positive electrode; the charging voltage was derived from the constant voltage output of the electrochemical workstation. Through experimental comparison, the charging voltage of 4 V and the charging time of 30 s turn out to have the best charging effect (Figure S16, Supporting Information). As shown in Figure 4d, the current density of the original device is about 4.2 µA cm^−2^; the instantaneous current density can reach 150 µA cm^−2^ after 30 s charging; the current density of the charged device is still about 6.35 µA cm^−2^ even after 24 h continuous discharge; the current density (4.66 µA cm^−2^) remains higher than that before charging (4.2 µA cm^−2^) after three times repeated charging. These facts prove that the passivation layer can be effectively reduced by charging, which also shows that the passivation layer is an important factor affecting the performance of the device.

To further demonstrate the effectiveness of the calcium chloride additive, a device cyclic discharge‐charge test was conducted. As shown in Figure 4e, the device was cyclically charged and discharged at 1 µA cm^−2^. The devices without additives exhibit unstable and relatively large voltage polarization during cycling. The devices fail earlier in the charging cycle at a small current density of 1 µA cm^−2^ and the discharge plateau decreases from the 8th cycle, with the maximum polarization at 2600 min, which may be due to the accumulation of oxide on the electrode surface, resulting in high polarization on the EGaIn surface. In contrast, devices containing additives can be cycled for up to 4800 min with reduced overpotential during cycling, showing stable cycling durability. This long‐lasting stability may be due to the additives that disrupt the accumulation of electrode oxides so as to allow the continuous diffusion of ions at the electrodes.

Then, the effect of applied charging voltage on the growth and disappearance of the passivation layer on the bottom electrode surface has been studied. First, the interface resistance changes of the device before and after charging were investigated using electrochemical impedance spectroscopy (EIS), as shown in Figure 4f. The internal resistance of the device decreases after charging, while the ionic conductivity increases (Figure S17, Supporting Information). The decreased internal resistance of the device is mainly due to the reduction of the oxide passivation layer under the applied voltage, resulting in faster ion transport and increasing conductivity inside the device. In order to eliminate the interference of the capacitance storage of the carbon material electrode, the EGaIn electrode was replaced by CNT, and then the device was charged and discharged. As shown in Figure S18, Supporting Information, the device does not show a visible increase in current density after charging. Therefore, the current change in the device after charging comes from the reduction of the electrode oxide rather than from the capacitive storage discharge of the carbon material.

Finally, the reversibility of electrode charging and discharging has been investigated. Figure 4g shows the SEM and EDS maps of the electrode reaction surfaces, respectively, after the first discharge process, after the first charge process, and after 40 discharge–charge cycles. The Ga content of the anode decreases from 75% to 71.71% after the first discharge, increases to 73.51% after the first charge, and remains almost constant (73.08%) after 40 cycles. To accurately assess the elemental changes in the anode, the Ga/In ratio is determined and the results are shown in Figure 4h. Since In is less active than Ga, Ga participates in the overall reaction, and the Ga − 3e^−^ ⇄ Ga^3+^ reaction occurs.^[^ ^28^ ^]^ Furthermore, the Ga/In ratio remains almost constant after 40 charging cycles (0.43 wt% decrease with respect to that after the first charging cycle), which indicates that the liquid metal electrode has excellent reversibility.

### 2.6 Applications of MEG

**Figure 5:**
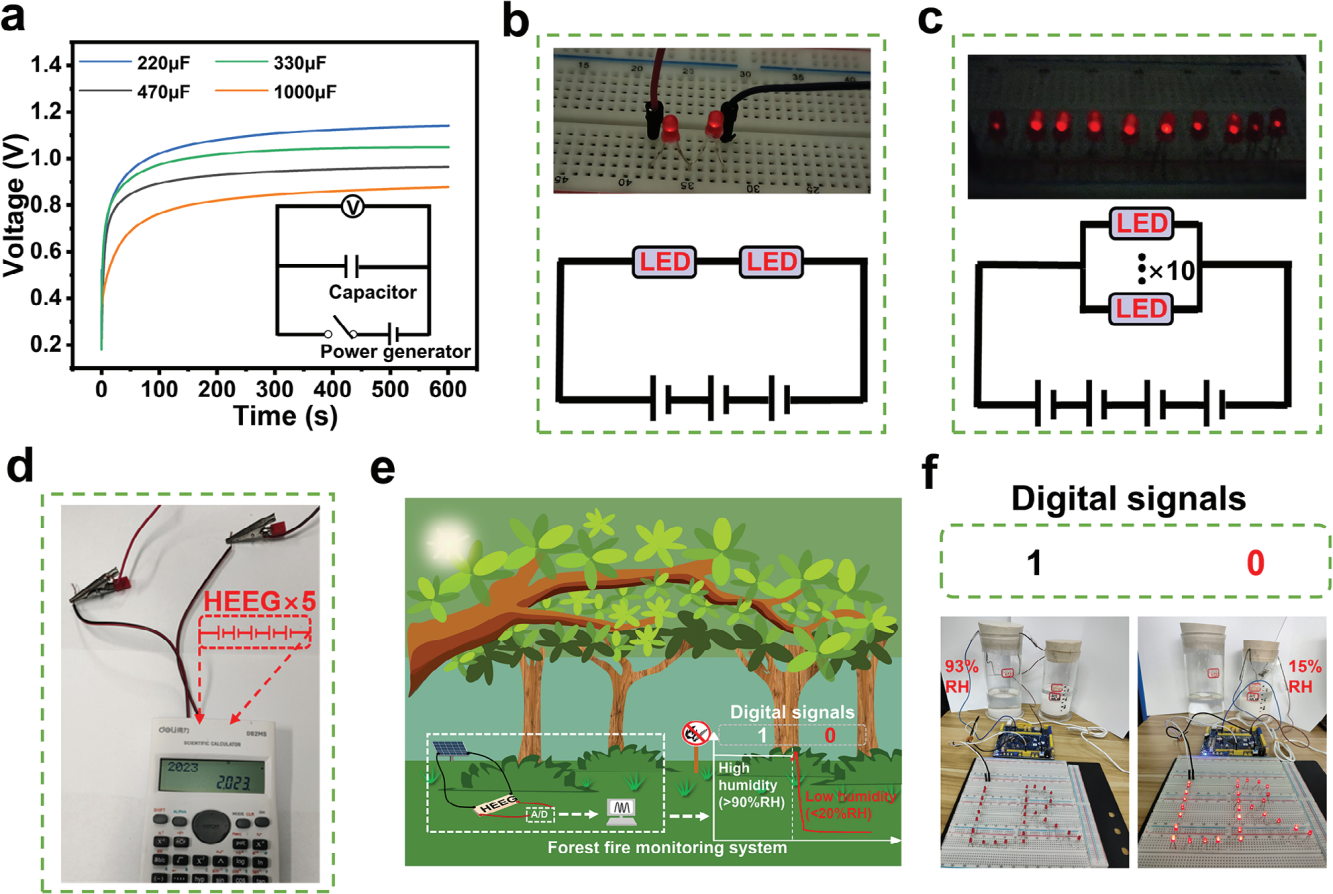
a) Voltage‐time curves of different commercial capacitors charging by a single MEG. b) Three devices in series are used to light two commercial LEDs in series. c) Four devices in series can light ten LEDs in parallel. d) Five devices in a series can light a commercial scientific calculator. e) Schematic diagram of the practical applications of the forest fire monitoring system. f) The demonstration experiment showing humidity sensing, at low humidity, the LED arrays could be lit.

The power provided by the MEG can be stored in commercial energy storage devices without the need for additional rectifiers. **Figure** **5**a shows the voltage‐time profile of a single device charging different commercial capacitors under moisture conditions. In addition, MEG can be used as a power source to supply power directly to commercial electronic devices. Three MEGs in series can illuminate two commercial red LEDs in series (Figure 5b). Four MEGs in series can illuminate ten commercial red LEDs in parallel (Figure 5c). Five MEGs in series can support the operation of a scientific calculator (Figure 5d). Apart from serving as a power supply, MEG's significant potential difference at high/low humidity can be taken as the basis for signal conversion. By setting the appropriate potential threshold, the potential output can be encoded as the digital signals “0” and “1.” Figure 5e shows a simulation of a high/low humidity control loop alarm. At high humidity (>90%RH), the feedback loop outputs a digital signal “1” to control the external circuit break, and once low humidity (<20%RH) is reached, the feedback loop outputs a digital signal “0” and the indicator lights up to indicate the low humidity state. Figure 5f shows the analog control circuit diagram (Video S2, Supporting Information). This method provides effective feedback on the humidity status of the device. By sensing changes in humidity, the device can be used for a wide range of applications such as forest fire alarms and agricultural greenhouse humidity monitoring.

## 3 Conclusion

In this work, we have introduced an electrode chemistry regulation strategy into the ionic diode‐type energy conversion structure, which demonstrates prolonged high‐power generation in ambient humidity. The electrode chemical regulation can be achieved by adding calcium chloride. This is essentially attributed to the destruction of the oxide passivation layer on the electrode surface. By introducing an appropriate amount of calcium chloride additive near the electrode, our device simultaneously satisfies the requirements of fast trapping of moisture molecules, high rectification ratio transport of ions, and sustained charge conversion at the ion‐electron coupling interface. Compared with those without additives, our devices have significantly improved both the duration of power generation and the output power. For example, a single device can deliver an open‐circuit voltage of ≈1 V and a peak short‐circuit current density of 350 µA cm^−2^.

The first‐principle calculations have been carried out to simulate the destruction process of the passivation film on the electrode surface by chloride ions. Indeed, the destruction of the passivation film provides a path for the ion‐electron coupling conduction at the electrode interface. In addition, the reduction of the passivation film on the electrode surface can be achieved by applying a voltage to the device, so as to achieve the purpose of reactivating the electrode and helping the device to recover its power generation performance. This also strongly proves that the passivation layer is an important factor affecting the performance of the device. After using calcium chloride additives, the power generation time of the device exceeds 1240 h.

Our work reveals the influence mechanism of electrode surface chemistry on moisture‐based power generation performance. It also provides a reliable solution for ultra‐long‐time humidity power generation, making the hydroelectric technology better match the long‐time energy supply needs of small smart devices.

## Conflict of Interest

The authors declare no conflict of interest.

## Supporting information

Supporting Information

Supplemental Video 1

Supplemental Video 2

## Data Availability

The data that support the findings of this study are available from the corresponding author upon reasonable request.
