# Supplementary material for: A Long Life Moisture‐Enabled Electric Generator Based on Ionic Diode Rectification and Electrode Chemistry Regulation
Source: Adv Sci (Weinh). 2024 Feb 14;11(15):2305530. doi: 10.1002/advs.202305530 (PMC11022712; doi:10.1002/advs.202305530)
Supplement: Supplementary file 1 — Supporting Information [file ADVS-11-2305530-s003.pdf]

ADVANCED SCIENCE
Open Access

Supporting Information

for *Adv. Sci.*, DOI 10.1002/advs.202305530

A Long Life Moisture-Enabled Electric Generator Based on Ionic Diode Rectification and Electrode Chemistry Regulation

Chunqiao Fu, Jian Zhou, Xulei Lu, Haochen Feng, Yong Zhang, Kedong Shang, Zhongbao Jiang, Yuming Yao, Qi-Chang He and Tingting Yang**

A long life moisture-enabled electric generator based on ionic diode rectification and electrode chemistry regulation

Chunqiao Fu[1], Jian Zhou[1], Xulei Lu[1], Haochen Feng[1], Yong Zhang[1], Kedong Shang[1], Zhongbao Jiang[1], Yuming Yao[1], Qi-Chang He*[,1,2], and Tingting Yang*[,1]

[1]Tribology Research Institute, School of Mechanical Engineering, Southwest Jiaotong University, Chengdu 610031, PR China

[2]Univ Gustave Eiffel, MSME, CNRS UMR 8208, F-77454 Marne-la-Vallée, France

*Corresponding authors. Email addresses: yangtingting@swjtu.edu.cn; qichang.he@u-pem.fr

Table of Contents

Experimental Procedures **3**
Results and Discussion **4**
Reference **12**
Author Contributions **13**

Experimental Procedures

Materials preparation: The AAO membrane with different pore size and area size (fixed thickness of ~47 μm) was purchased from Shenzhen Topmembranes Technology Co, Ltd., China. CNT films were grown by chemical vapor deposition (CVD). The EGaIn was purchased from Dongguan Wochang Metal Products Co., Ltd. The Calcium chloride was purchased from Sinopharm Group Chemical Reagent Co., Ltd.

Fabrication process of the device: First, the commercial AAO film was treated with oxygen plasma (MIT Corporation PCE-6) for a period of time (180 seconds, 35 W power) for standby. Second, a pipette gun was used to drop 2 μl of calcium chloride solution on the upper surface of AAO. And the sample was spin-coated (10s, 200r/min) to ensure that the calcium chloride was evenly deposited. Then the upper surface of AAO was turned over to the lower surface after spin coating. Third, the liquid metal, which acts as the bottom electrode, was scraped onto an appropriately sized glass slide to make it evenly distributed. The AAO film lower surface was attached to the surface of the liquid metal. And carbon fiber wire was used to conduct liquid metal for electrical output. Then, the CNT film (~600 nm for thickness) was transferred on the upper side of the AAO film as the top electrode, a conductive carbon tape (Nissin SEM double-sided carbon conductive tape) was used to connect the conductive carbon fiber to the top electrode of the device. Finally, the prepared device was treated with high power (35 W) oxygen plasma for a period of time such as 180 s.

Electrical Output Measurement: The device output V_{OC} and I_{SC} were recorded by electrometer (Keithley 6514) and digit multimeter (Keithley DMM7510). The Tafel Plot, electrochemical impedance spectroscopy (EIS) and chronopotentiometry of the device were scanned using CHI electrochemical analyzer (CHI760E, CH Instruments, Inc, US). Power analog input for device charging is derived from the Multi-Potential Steps module of the CHI760 electrochemical analyzer.

Material Characterization: Scanning electron microscope (SEM) (JSM 7800 F, Japan) was used to characterize the morphology of CNT, GaIn and AAO, and X-ray energy dispersion spectroscopy (EDS) (OXFROD X-Max 80) was used to characterize the elemental composition of the GaIn Oxides. X-ray photoelectron spectroscopy (Thermo

Scientific K-Alpha) was used to analyze the surface element composition of CNT and achieve semi-quantitative analysis of their element content.

Results and Discussion

1. Analysis of power generation in the MEG

(1) Voltage Determinants

The total voltage (V_0) measured is divided into two parts: the built-in potential (V_B) generated by the ion diode and the redox potential (V_{REDOX}) generated by the redox reaction at the electrode interface, both of which satisfy the Ernst equation:

$$V_0 = V_B + V_{REDOX} \quad (1)$$

$$V_{REDOX} = V^0 + \frac{RT}{nF} ln \frac{\prod \alpha_{REACTANT}^{v}}{\prod \alpha_{PRODUCT}^{V'}} \quad (2)$$

Where V^0, v, v', n, R, T, and F are, respectively, standard electromotive force for redox reactions, the stoichiometric numbers of reactants and products, the number of electrons participating in the reaction, the molar gas constant, temperature, and Faraday constant. $\alpha_{REACTANT}$, $\alpha_{PRODUCT}$ are the activity, and the physical meaning of the activity is the effective concentration.

(2) Current Determinants

The short-circuit current (J_{SC}) depends on the electrochemical reaction rate of the electrode. According to chemical kinetics, reaction rate v and reaction activation energy Δ The relationship between ΔG is represented as:

$$V = kc \exp(-\frac{\Delta G}{RT}) \quad (3)$$

where k and c are the pre-exponential factor and the reactive particle concentration, respectively.

2. Specific calculation steps for the donor density N_D

The donor density N_D of the passive films can be estimated from the slope of linearly fitted

Mott-Schottky plots based on the following equation:

$$C^{-2} = \frac{2}{\varepsilon\varepsilon_0 e N_{\mathrm{D}}}\left(E - E_{FB} - \frac{KT}{e}\right) \tag{4}$$

at room temperature, $\frac{KT}{e} \approx 25\mathrm{mv}$, which can be negligible.

$$C^{-2} = \frac{2}{\varepsilon\varepsilon_0 e N_{\mathrm{D}}}(E - E_{FB}) \tag{5}$$

The slope of the curve is considered as *S*.

$$S = \frac{C^{-2}}{E-E_{FB}} \tag{6}$$

$$N_{\mathrm{D}} = \frac{2}{e\varepsilon\varepsilon_0 S} \tag{7}$$

where ε is the dielectric constant of passive films, here ε is taken as 10[1]. ε_0 is the vacuum permittivity constant (8.85419×10^{-12} F/m). *e* is the elementary charge (1.6×10^{-19}C), N_{D} is the donor density of the Passive Films, *K* is the Boltzmann's constant (1.38×10^{-23} J/K), $E_{\mathbf{FB}}$ is the flat band potential, *T* is the absolute temperature (298 K), and $\frac{KT}{e}$ is about 25 mV at room temperature, which can be negligible. The slope of the curve can be derived from the image. According to Equation (4), we can calculate N_{D}.

Table S1. Values of N_{D} at different chloride ion concentrations

Concentration (M)	0.1	0.2	0.3	0.4
Slope	8.59	4.7	4.1	2.84
N_{D} (10^{19} cm^{-3})	0.16	0.3	0.34	0.496

3. Description of the specific parameters of DFT

We used the DFT as implemented in the Vienna Ab initio simulation package (VASP) in all calculations. The exchange-correlation potential is described by using the generalized gradient approximation of Perdew-Burke-Ernzerhof (GGA-PBE). The projector augmented-wave (PAW) method is employed to treat interactions between ion cores and

valence electrons. The plane-wave cutoff energy was fixed to 500 eV. Given structural models were relaxed until the Hellmann–Feynman forces smaller than -0.02 eV/Å and the change in energy smaller than 10^{-5} eV was attained. During the relaxation, the Brillouin zone was represented by a Γ centered k-point grid of 2×2×1. A vacuum layer of around 20 Å was added in the direction perpendicular to the surface to eliminate the spurious interlayer interaction. A 3×3 GaOOH (001) supercell was built for the calculations.

Table S2. Performance parameter comparison of moisture-enabled electric generators.

Material	RH/ΔRH (%)	V_{OC} Voltage (V)	J_{SC} Current density (μA cm^{-2})	$P_{max}=V_{max}\times J_{max}$ (μW cm^{-2})	Time (h)	Ref
**Cycling fabric**	40±5	0.85	256	217.6	720	[2]
**Polymer dendritic colloids**	80	1.45	16.7	24.2	120	[3]
**Polyelectrolyte films**	85	1.38	4	5.52	258	[4]
**Fluorinated oxidized graphene**	60	0.75	12	9	11.1	[5]
**CNT/AAO**	93	1.1	11.3	12.43	720	[6]
**PA-gelated hydrogel**	65	0.88	240	211.2	1000	[7]
**CNT/AAO/$CaCl_2$**	93	1.0	350	350	1240	This work

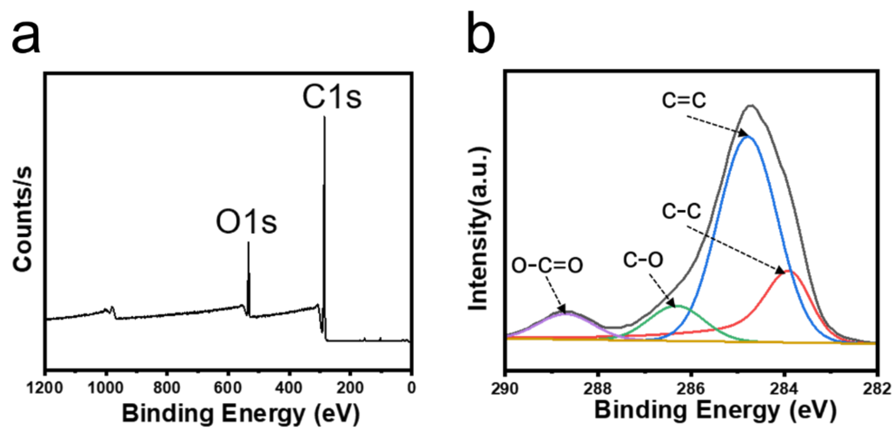

Supplementary Figure 1. XPS spectrum of the CNT film. (a) The XPS spectra of CNT film. (b) Fine spectroscopy of XPS for CNT.

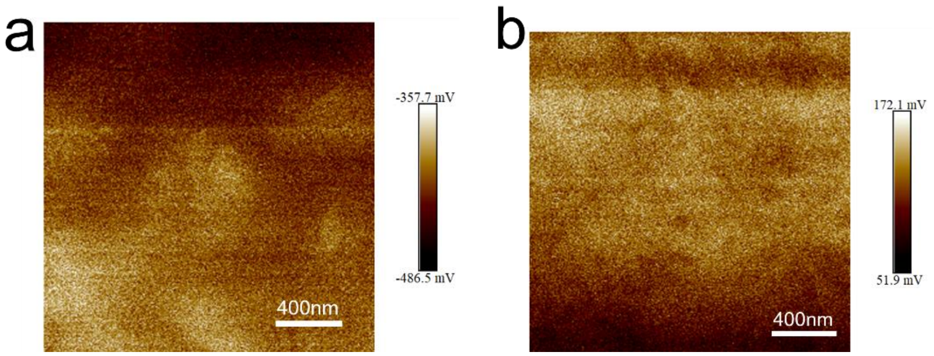

Supplementary Figure 2. KPFM of the CNT film. a) Surface potential of CNT film by KPFM. b) Surface potential of CNT loaded on the AAO surface by KPFM.

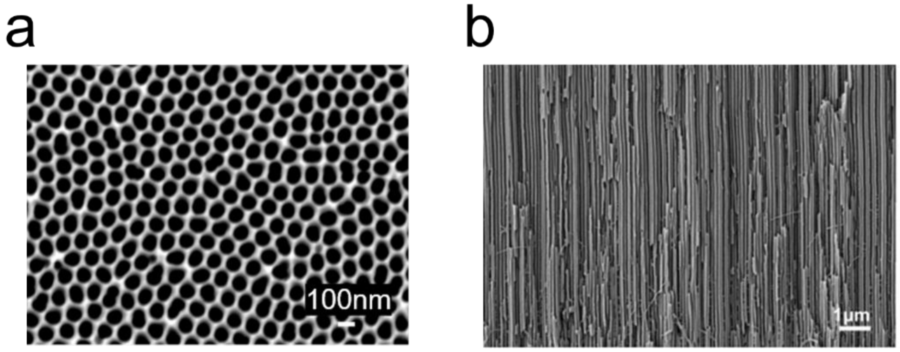

Supplementary Figure 3. SEM morphology of AAO. (a) Top view of AAO membrane. Regularly arranged through pores facilitate the adsorption and transport of water molecules. (b) Cross-sectional SEM images of AAO membrane.

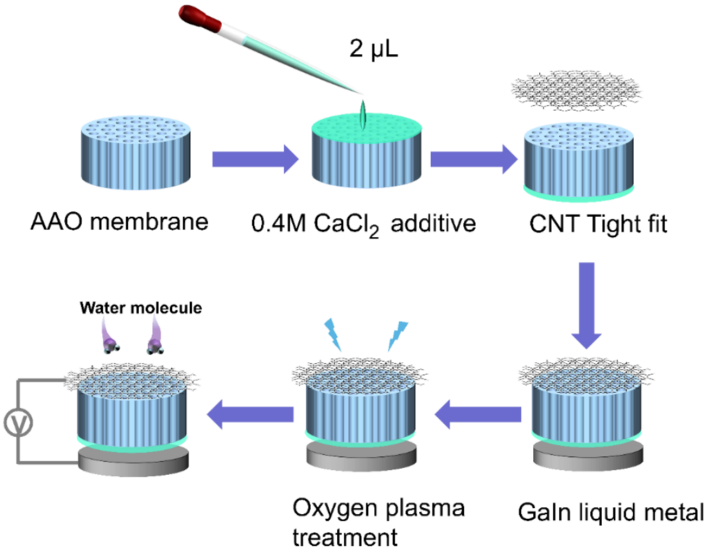

Supplementary Figure 4. The process of MEG fabrication. Detailed process is illustrated in the method part.

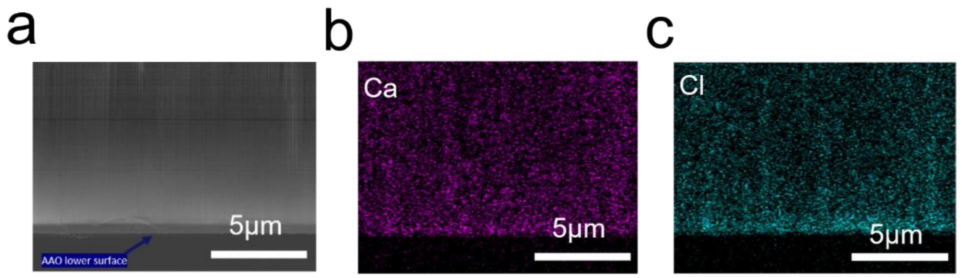

Supplementary Figure 5. Distribution of calcium chloride ions in the nanopore channel. (a) AAO cross-sectional SEM images. (b) EDS spectrum of calcium element in the nanopore channel. (c) EDS spectrum of chlorine element in the nanopore channel.

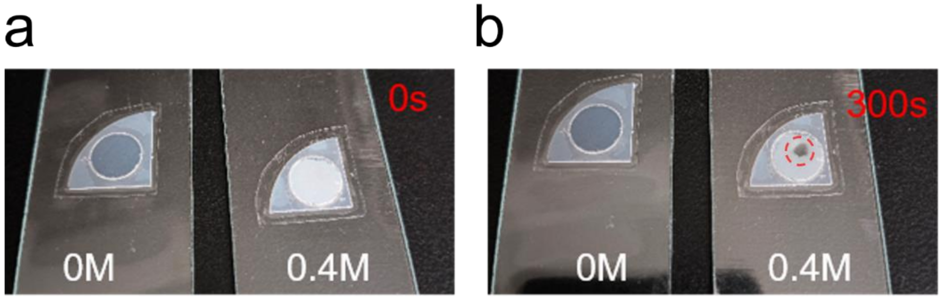

Supplementary Figure 6. AAO surface water absorption test. (a) Water absorption of AAO at different concentrations of calcium chloride at 0 seconds. (b) Water absorption of AAO under different concentrations of calcium chloride after 300 seconds.

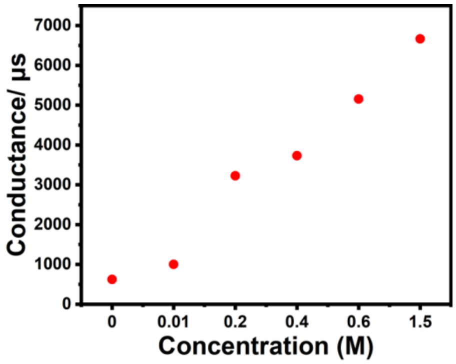

Supplementary Figure 7. Transmembrane ionic conductance for different additive concentrations. The ionic conductivity was calculated at different concentrations, the higher the concentration, the greater the electrical conductivity.

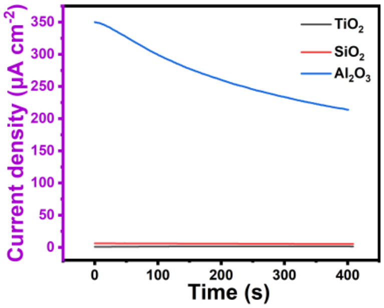

Supplementary Figure 8. Current density of MEGs using different oxides. By replacing AAO with nanoporous films with different electronegativity to avoid PN junction formation, the current densities of the alternative devices were tested.

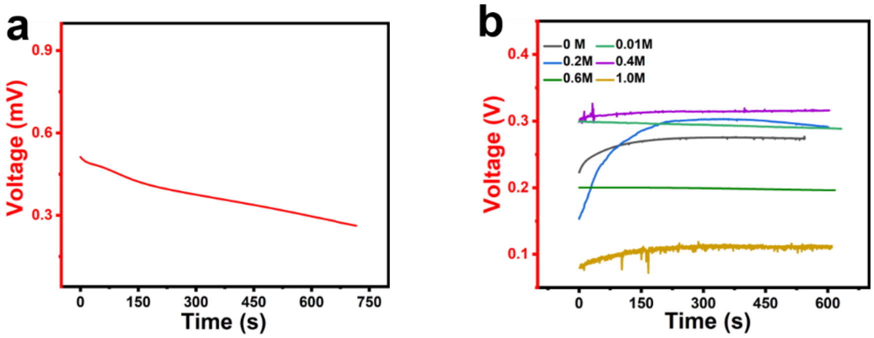

Supplementary Figure 9. Device open circuit voltage after using carbon material as the lower electrode. a) The voltage of the device when the upper and lower electrodes are simultaneously exposed to air. b) Open-circuit voltage of the device at different concentration additives (lower electrode seal). The open circuit voltage has the highest

value at a additive concentration of 0.4M.

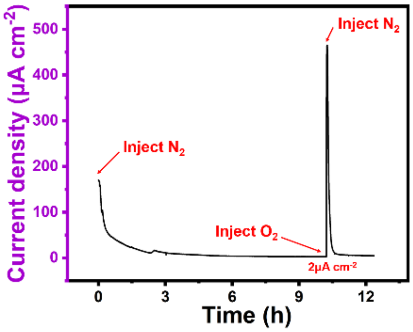

Supplementary Figure 10. Device current density under different gas conversions. When nitrogen is introduced into the sealed chamber, the current density gradually decreases to a minimum of 2 μA cm^{-2} (with a small amount of residual oxygen still in the chamber). When oxygen is then rapidly introduced, the current density rapidly climbs to 450 μA cm^{-2}.

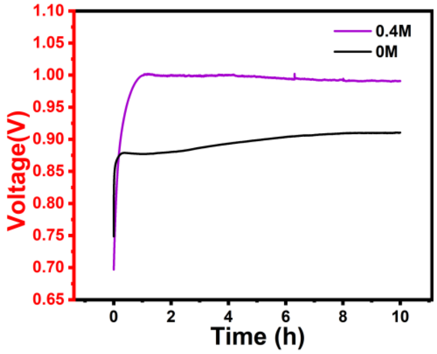

Supplementary Figure 11. Voltage curve of HEEG with and without calcium chloride. After 10 hours of continuous discharge, the voltage of the device without calcium chloride additive is about 0.9V, and the voltage of the device with 0.4M calcium chloride additive is about 1V.

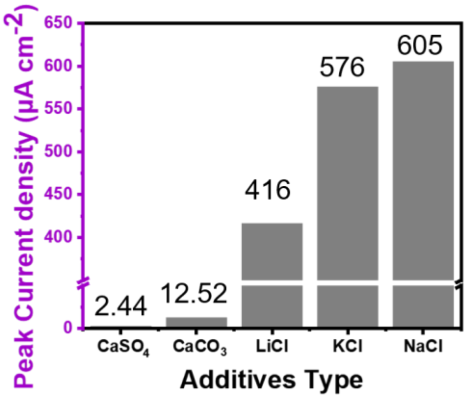

Supplementary Figure 12. Chlorine ion effectiveness experiment. The performance tests were conducted separately using chlorine-containing compounds and calcium-containing compounds, and the experimental results showed that chloride ions play a dominant role in the output process of the device.

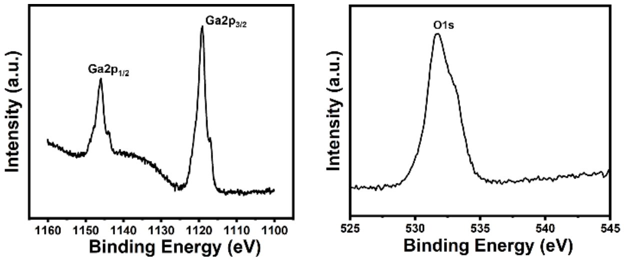

Supplementary Figure 13. **High-resolution XPS spectra of GaOOH.**

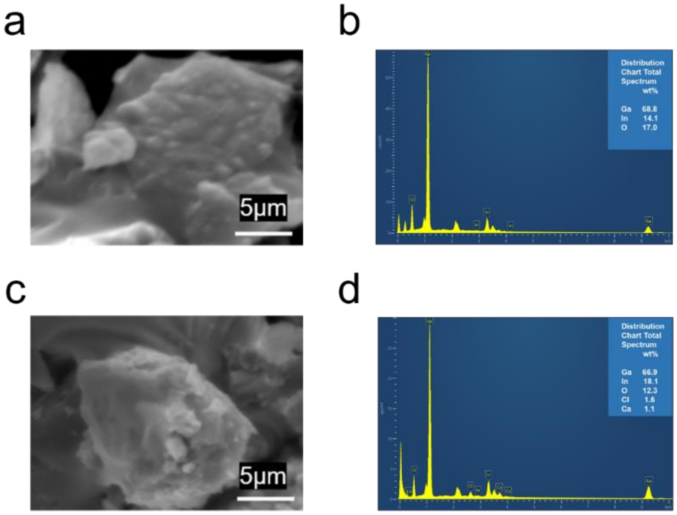

Supplementary Figure 14. EDS images of the electrode reaction interface with and without calcium chloride additives. (a) SEM images and (b) corresponding EDS images of electrode oxides in the absence of calcium chloride. (c) SEM images and (d)

corresponding EDS images of electrode oxides containing calcium chloride. The mass fraction of elemental oxygen without calcium chloride additive is 17%. The mass fraction of elemental oxygen when containing calcium chloride additive is 12.3%.

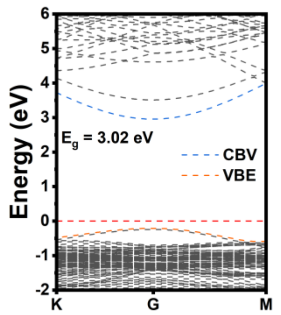

Supplementary Figure 15. Original energy band of GaOOH. The initial value of the band gap is 3.02eV.

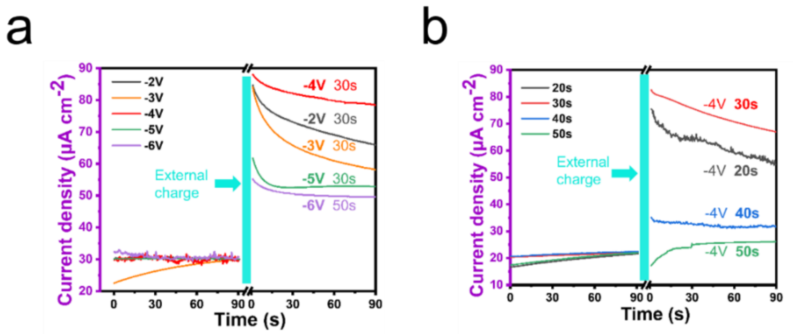

Supplementary Figure 16. Charging voltage and charging time selection. (a) Current density at different charging voltages. (b) Current density at different charging times.

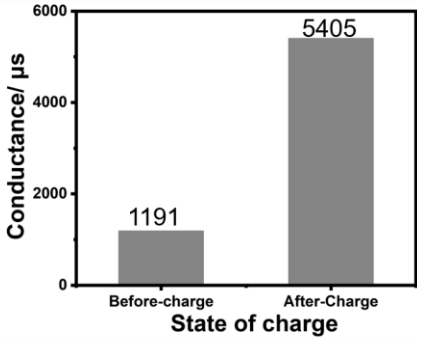

Supplementary Figure 17. Change of ionic conductivity before and after charging. The ionic conductivity of the device before charging is 1191μs. The conductivity after charging is 5405μs.

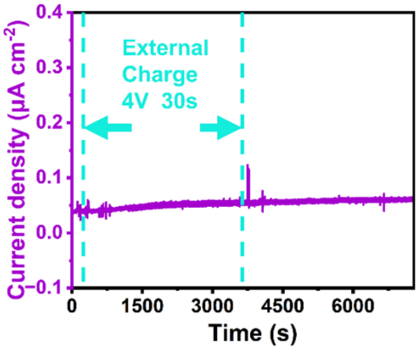

Supplementary Figure 18. Charge and discharge performance of double carbon material electrode. Current output after charging the device at 4Vand 30s. The device in which the EGaIn electrode is replaced by CNT has almost no discharge capability after charging, and the current density of the second discharge is 0.13μA • cm^{-2}.

Reference

[1] M. Passlack, N. Hunt, E. Schubert, G. Zydzik, M. Hong, J. Mannaerts, R. Opila, R. Fischer, *Applied physics letters* **1994**, *64*, 2715-2717.
[2] Y. Hu, W. Yang, W. Wei, Z. Sun, B. Wu, K. Li, Y. Li, Q. Zhang, R. Xiao, C. Hou, *Science Advances* **2024**, *10*, eadk4620.
[3] X. Zhang, Z. Dai, J. Chen, X. Chen, X. Lin, S. Yang, K. Wu, Q. Fu, H. Deng, *Energy & Environmental Science* **2023**, *16*, 3600-3611.
[4] H. Wang, Y. Sun, T. He, Y. Huang, H. Cheng, C. Li, D. Xie, P. Yang, Y. Zhang, L. Qu, *Nature Nanotechnology* **2021**, *16*, 811-819.
[5] K. Fan, X. Liu, Y. Liu, Y. Li, X. Liu, W. Feng, X. Wang, *Nano Energy* **2022**, *91*, 106605.
[6] Y. Zhang, T. Yang, K. Shang, F. Guo, Y. Shang, S. Chang, L. Cui, X. Lu, Z. Jiang, J. Zhou, *Nature Communications* **2022**, *13*, 3484.
[7] S. Yang, X. Tao, W. Chen, J. Mao, H. Luo, S. Lin, L. Zhang, J. Hao, *Advanced Materials* **2022**, *34*, 2200693.

Author Contributions

T. Y. and C.F. designed the research. T.Y. and Q.C.H. directed this research. T.Y., C.F. and Q.C.H. wrote the paper. C.F. conducted the majority of the experimental work. T.Y. proposed mechanisms. F.H. provided assistance in theoretical

calculations. X. L., Y. Z. analyzed data and discussed results. J. Z. and Z. J. tested the SEM of AAO film. X. L., and K. S., assisted in setting up test equipment.
